# *De novo* transcriptome assembly database for 100 tissues from each of seven species of domestic herbivore

**DOI:** 10.1038/s41597-024-03338-5

**Published:** 2024-05-11

**Authors:** Yifan Wang, Yiming Huang, Yongkang Zhen, Jiasheng Wang, Limin Wang, Ning Chen, Feifan Wu, Linna Zhang, Yizhao Shen, Congliang Bi, Song Li, Kelsey Pool, Dominique Blache, Shane K. Maloney, Dongxu Liu, Zhiquan Yang, Chuang Li, Xiang Yu, Zhenbin Zhang, Yifei Chen, Chun Xue, Yalan Gu, Weidong Huang, Lu Yan, Wenjun Wei, Yusu Wang, Jinying Zhang, Yifan Zhang, Yiquan Sun, Shengbo Wang, Xinle Zhao, Chengfang Luo, Haodong Wang, Luoyang Ding, Qing-Yong Yang, Ping Zhou, Mengzhi Wang

**Affiliations:** 1 State Key Laboratory of Sheep Genetic Improvement and Healthy Production, Xinjiang Academy of Agricultural Reclamation Sciences, Shihezi, 832000 P. R. China; 2 College of Animal Science and Technology, Yangzhou University, Yangzhou, 225009 P. R. China; 3 College of Life Science, Guizhou University, Guiyang, 550025 P. R. China; 4 Hubei Key Laboratory of Agricultural Bioinformatics and Hubei Engineering Technology Research Center of Agricultural Big Data, College of Informatics, Huazhong Agricultural University, Wuhan, 430070 P. R. China; 5 College of Animal Science and Technology, Hebei Agricultural University, Baoding, 071033 P. R. China; 6 College of Life Science, Linyi University, Linyi, 276005 P. R. China; 7 UWA Institute of Agriculture, The University of Western Australia, Perth, WA 6009 Australia

**Keywords:** Gene expression, Bioinformatics

## Abstract

Domesticated herbivores are an important agricultural resource that play a critical role in global food security, particularly as they can adapt to varied environments, including marginal lands. An understanding of the molecular basis of their biology would contribute to better management and sustainable production. Thus, we conducted transcriptome sequencing of 100 to 105 tissues from two females of each of seven species of herbivore (cattle, sheep, goats, sika deer, horses, donkeys, and rabbits) including two breeds of sheep. The quality of raw and trimmed reads was assessed in terms of base quality, GC content, duplication sequence rate, overrepresented k-mers, and quality score distribution with FastQC. The high-quality filtered RNA-seq raw reads were deposited in a public database which provides approximately 54 billion high-quality paired-end sequencing reads in total, with an average mapping rate of ~93.92%. Transcriptome databases represent valuable resources that can be used to study patterns of gene expression, and pathways that are related to key biological processes, including important economic traits in herbivores.

## Background & Summary

Herbivores have been critical for food security since the late Holocene and remain an important contributor to that security^1^. There are around four billion domestic herbivores on the planet, representing animals from diverse species and breeds, with different species and breeds adapted to, and therefore farmed, in different environments, even marginal lands^2^. The species or breed of domestic herbivore that is found in an agricultural ecosystem is adapted to that system because of its physiological adaptation to that ecosystem in terms of characteristics such as growth rate, mature size, reproductive strategy, and/or digestive system (Table 1). However, some complex phenotypes, such as specific sexual behaviours, are common to different species of domestic herbivores. For example, “flehmen” behaviour, the typical behaviour of a male exposed to the urine of a female, is shared by many herbivores including the seven species used here^3^. Conversely, the timing of the breeding season can be quite different between species. A response to photoperiod drives sheep to breed during winter (short-day breeders) while the same photoperiodic signal causes horses to breed during summer (long-day breeders), and some other species breed all year round (Table 1). Breeds within a species generally occupy different ecological niches because they have been selected, either naturally or artificially, to survive and reproduce within that particular environment. A further understanding of the interaction between genetics and the environment on the expression of phenotype is essential to improve the management of domestic herbivores.

**Table 1:** Summary of facts on the seven species included in the present study.

Species	Worldwide value (US$ millions)^a^	Adult Live weight (kg)^b^	Reproduction^b^			Type of digestive system^b^	Agricultural use^b^				
			Sexual maturity (month)	Reproductive strategy	Litter size		Meat	Milk	Fibre	Hide	Power
*Horse*	60	230–900	Female: 30 Male: 32	Seasonal breeder long day	1	Hindgut fermenter	•	•			•
*Donkey*	53	180–225	Female: 18 to 24 Male: 9 to 24	Seasonal breeder long day	1	Hindgut fermenter	•	•			•
*Cattle*	1,529	500–1300	Female: 12–18 Male: 12	Year round	1	Ruminant	•	•		•	•
*Goat*	1,111	10–110	Female: 13 Male: 23	Seasonal breeder short day	1–3	Ruminant	•	•	•	•	
*Sheep*	1,284	20–200	Female: 18 Male: 30	Seasonal breeder short day	1–3	Ruminant	•	•	•	•	
*Deer*	5	60–200	Female: 16–18 Male: 16–18	Seasonal breeder short day	1	Hindgut fermenter	•			•	
*Rabbit*	0.172	1.5–2.5	Female: 8 Male: 8	Year round	1–14	Hindgut fermenter	•		•		

^a^Worldwide value from FOASTAT (https://www.fao.org/faostat/,accessed on 16 August 2023), except for deer^23^.

^b^Data on adult live weight, reproduction, digestive system and agriculture uses were obtained from ABW (Animal Biodiversity Web) University of Michigan’s animal diversity web (https://animaldiversity.org/) and AnAge Database of Animal Ageing and Longevity (https://genomics.senescence.info/species/index.html). Both were accessed on 16 August 2023.

While the study of variation or mutation in the genome is informative, most complex phenotypes, such as behaviour, are encoded by an interacting suite of numerous genes, potentially involving hundreds of genes^4^. Comparative transcriptomics is a powerful tool that can be used to explore the interaction between genotype and environment in the context of biological adaptation and the potential limits of a species^4^. Using a combination of genomics and transcriptomics was first proposed by the Functional Annotation of Animal Genomes (FFANG) Consortium to identify the genes that code for proteins that are involved in critical production traits in farm animals^5^. Transcriptomic atlases were generated for common domestic animals such as pigs, cattle, sheep, and chickens^6–10^ as well as other species of herbivores such as donkey^11^, water buffalo^12^, and goat^13^. While these published transcriptomic atlases of herbivores are valuable, they are published in independent databases, with no standard protocols for sample collection, RNA sequencing, or data processing. While the transcriptomic database for cattle includes 51 tissues^8^, most of the other published transcriptomic atlases contain a relatively small number of tissues (ranging from 12 to 25). Moreover, while the transcriptomic data from a particular tissue is informative about the transcriptome of that particular organ or tissue, it is not necessarily representative of the entire transcriptome of every organ or tissue. Therefore, it is essential to obtain samples from specific regions within organs that are functionally heterogeneous, such as the brain^7,8^. Similarly, it could be argued that a complete transcriptome should include representative tissues from each of the ten main organ systems, and that the collection and analysis protocal should be the same for each. To our knowledge, none of the transcriptome atlases mentioned above analysed samples from different regions of heterogenous tissues or organs from each of the ten main organ systems using a standard protocol^7,8^.

**Fig. 1:**
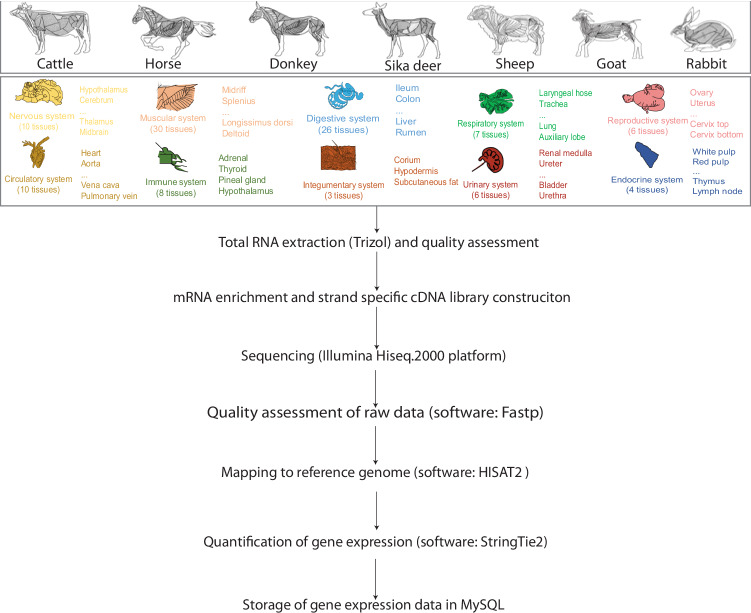
Schematic overview of the data collection and the quality validation of the herbivore RNA-seq database.

**Fig. 2:**
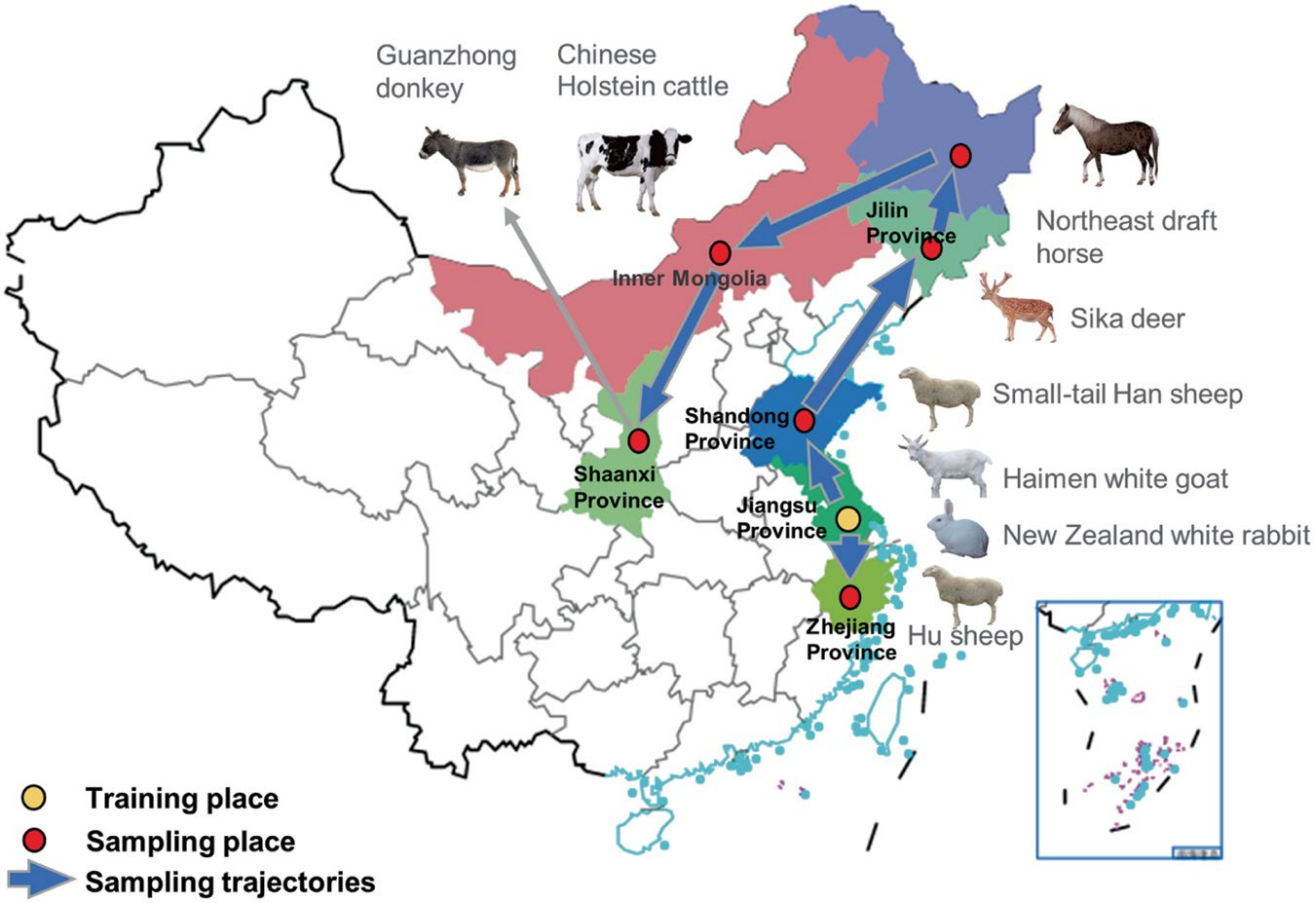
An overview of the sampling location and geographic distributions of the horse, the donkey, the rabbit, the deer, the goat, cattle, and sheep in China.

With the current study we aimed to generate a public raw RNA-seq database that will facilitate the comparison of gene expression profiles between different tissues from seven species of domestic herbivores that have important economic value (Fig. 1). The seven species were the horse, the donkey, the rabbit, the deer, the goat, cattle, and sheep. Two breeds of sheep, the Hu and the Han sheep, were included because of their economic relevance and specific geographic distributions in China (Fig. 2a). The transcriptome was analysed in organs and tissues from the ten organ systems, and regions within heterogenous organs (Fig. 1). This database can be used primarily to further identify the genetic foundations of economically significant traits and open novel pathways to genetic improvement. Secondarily, this database could facilitate further understanding of the evolutionary processes that have taken place in herbivores during their domestication and the interaction between genetics and environment on the expression of similar and different phenotypes.

## Methods

All animal experiments were carried out in accordance with the ARRIVE guidelines and were approved by the Animal Ethics Committee of Yangzhou University under approval number RA202203-046. The experimental processes involved in the data collection and quality validation are shown in Fig. 1.

### Data collection

The RNA-seq data were obtained using transcriptome sequencing from between 100 and 105 tissue samples that were dissected from two healthy juvenile female individuals of eight domestic herbivores, representing seven species and including two breeds of sheep (Table S1). The seven species were Chinese Holstein cattle (*Bos taurus*) samples), Haimen white goat (*Capra hircus*), Northeast draft horse (*Equus caballus*), Guanzhong donkey (*Equus asinus*), sika deer (*Cervus nippon*), New Zealand white rabbit (*Oryctolagus cuniculus*), and two breeds of the domestic sheep (*Ovis aries*), the Hu sheep and the Small-tail Han sheep. The tissue samples were selected to cover the ten organ systems (Table S1): integumentary system, muscular system, digestive system, respiratory system, urinary system, reproductive system, circulatory system, immune system, nervous system, and endocrine system^14^. Tissue samples were collected from all organs and regions within heterogenous organs within 15 minutes after death by overdose of propofol (H20051843, Guangdong Jiabo pharmaceutical Co. Ltd. Qingyuan China).

#### RNA extraction and sequencing

Total RNA was extracted from each tissue sample using Trizol (Qiagen, Germany) and from each blood sample using QIAamp RNA Blood Mini Kit (Qiagen). Contamination from genomic DNA was removed using DNase (New England Biolabs, USA). The quality and concentration of RNA was assessed using the Agilent 2100 RNA 6000 Nano Kit (Agilent Technologies, Germany). Construction of the transcript library, clustering, and sequencing were performed at the Novogene Bioinformatics Institute (Wuhan, China). Briefly, a total of 3 µg total RNA of each sample was purified using poly-T oligo-linked magnetic beads (Invitrogen, USA). The RNA strands were subsequently fragmented with divalent cations in NEB First Strand Synthesis reaction buffer (NEB, USA), followed by the synthesis of first and second strand cDNAs. The cDNAs were sequenced using the Illumina Hiseq. 2000 platform, generating 100 bp paired-end reads.

#### Quality control and processing of RNA-seq data

Fastp software was used to check the read quality of the RNA-seq and to filter out any low-quality reads^15^. Single-end and paired-end reads were then mapped to the reference genome using HISAT2 (v2.2.1) (Table 2)^16^, and the results were sorted and converted to BAM format using SAMtools^17^. The reference genome of deer, downloaded from RGD v2.0^18,19^, and the reference genomes of other species, from the NCBI database, were used for gene annotation. Data on gene expression were quantified using StringTie2^20^. The expression level of each gene was normalized as both fragments per kilobase of exon per million mapped fragments (FPKM) and as transcripts per kilobase of exon model per million mapped reads (TPM). The sample-sample relationship in each of the seven species of herbivore was assessed with a Pearson correlation coefficient test using the gene expression data, and the results are reported in the supplementary figures.

**Table 2:** Descriptive summary of the newly generated profiles of transcriptome sequencing that were based on 110 organs/tissues from each of eight species/breeds and the annotation from databases that are available in the public domain.

Species	Breed	Number of samples	Number of reads	Aligned ratio (%)	Number of genes detected in present study	Proportion of protein-coding to non-coding genes	Reference genome	Ref	Annotation database
*Horses*	Northeast draft horse	204	5,665,395,789	96.06	33,091	1.76	EquCab3.0	^24^	*Equus caballus* RefSeq Annotation Release 103^25^
*Donkeys*	Guanzhong donkey	202	7,166,740,391	91.7	38,516	1.28	ASM1607732V2	^26^	*Equus asinus* Annotation Release 101^27^
*Cattle*	Chinese Holstein cattle	210	7,182,117,084	95.69	34,801	1.52	ARS-UCD1.2	^28^	*Bos taurus* Annotation Release 106^29^
*Goats*	Haimen white goat	208	7,082,253,582	96.4	28,920	2.48	ARS1	^30^	*Capra hircus* Annotation Release 102 ^31^
*Sheep*	Hu sheep	210	6,918,363,418	96.25	33,800	1.69	ARS-UI_Ramb_v2.0,	^32^	*Ovis aries* Annotation Release 102^33^
				90.41	29,298	2.34	Oar_v4.0	^34^	*Ovis aries* Annotation 104^35^
*Sheep*	Small-tail Han sheep	198	6,489,163,398	95.18	33,800	1.69	ARS-UI_Ramb_v2.0,	^32^	*Ovis aries* Annotation Release 102 ^33^
				90.17	29,298	2.34	Oar_v4.0	^34^	*Ovis aries* Annotation 104^35^
*Sika deer*	Sika deer	210	6,378,047,294	92.85	*	*	MHL_v1.0	^36^	*
*Rabbits*	New Zealand white rabbit	200	7,237,287,013	86.94	29,347	2.24	OryCun2.0	^37^	*Oryctolagus cuniculus* Annotation Release 101^38^

*The annotation for MHL_v1.0 contains a total of 21,449 protein-coding genes and the exon number per gene was 9.29^18,19^.

## Data Records

Raw RNA-Sequence reads of 1,642 tissues of seven species of herbivores (an average read mapping rate of 93.92%, Table 2) have been deposited in the NCBI Sequence Read Archive (SRA) database under the NCBI project (https://www.ncbi.nlm.nih.gov/bioproject/) with an accession number of PRJNA1017964^21^.

A hierarchical coding system was used to assign a unique code to each sample (Table S1) similar to Medical Subject Headings (MeSH)^22^. Each code comprises four levels. Level 1 indicates the organ system and starts with the letter “A” followed by a number between 01 and 10, representing the 10 organ systems. Level 2 comprises of two digits that represent the organ within the organ system. Level 3 comprises of three digits that represent the tissue within the organ. Level 4 comprises of two digits that represent the region within the tissue. The same codes are used with each species to facilitate the retrieval and analysis of both data generated for the present project and public RNA-Seq data.

## Technical Validation

### Quality control of tissue collection

The quality of the tissue that were collected for RNA sequencing in this study was optimised by using a very experienced team of neurobiologists, animal physiologists, veterinarians, and biologists to perform rapid sampling of the 100 to 105 tissues from each of the seven species and two breeds. Each sampling session was performed by a team of at least 15 people, so that all the 100–105 tissue samples were snap frozen in liquid nitrogen within 15 minutes after the animal was declared dead.

### Quality validation of RNA-Seq data

**Fig. 3:**
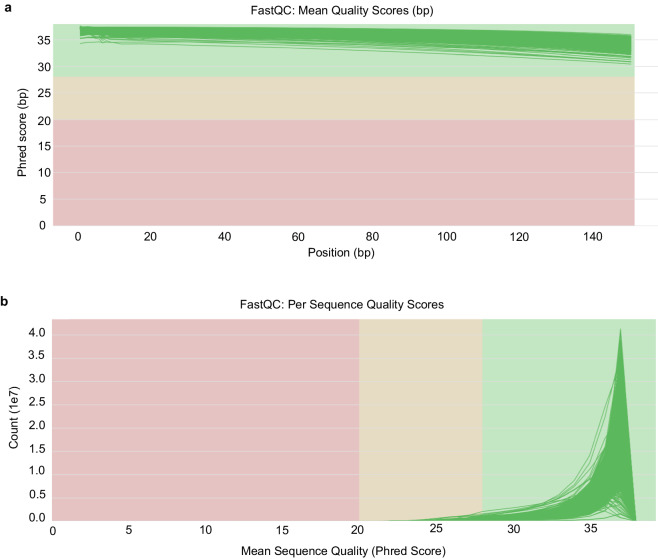
Sequence quality assessment metrics of filtered and trimmed RNA-Seq data using MultiQC. (**a**) The mean quality scores across each base position, (**b**) quality score distribution over all sequences.

The sequencing depth of the tissue samples from Haimen white goat, sika deer, Guanzhong donkey, Northeast draft horse, New Zealand white rabbit, Chinese Holstein cattle, Hu sheep, and Small-tail Han sheep are reported in Table S1. The minimum and mean sequencing depths were 1.94 and 3.49 in Haimen white goat, 2.26 and 3.65 in sika deer, 2.40 and 4.37 in Guanzhong donkey, 2.32 and 3.32 in Northeast draft horse, 2.06 and 3.96 in New Zealand white rabbit, 2.14 and 3.77 in Chinese Holstein cattle, 2.10 and 3.79 in Hu sheep, and 1.24 and 3.75 in Small-tail Han sheep. For the newly-generated RNA-seq datasets, the samples with low quality RNA and cDNA library were removed from the transcriptome catalogue. The quality of raw and trimmed reads was assessed in terms of base quality, GC content, duplication sequence rate, overrepresented k-mers, and quality score distribution using FastQC (http://www.bioinformatics.babraham.ac.uk/projects/fastqc). MultiQC software was used to aggregated and analysed the quality control metrics of all samples. Here, the quality report of newly generated RNA-seq data generated by MultiQC software showed that quality scores per base were high with phred score above 30 and most of sequences were high-quality with mean quality scores above 30 (Fig. 3). Next, high-quality filtered and trimmed RNA-Seq data was aligned to the reference genome of the corresponding species (Table 2). The alignment results indicated that the average aligned ratio of goat, sika deer, donkey, horse, rabbit, cattle, sheep were 94.35%, 91.84%, 93.28%, 93.11%, 87.40%, 94.91%, 95.57%, respectively (Table S1), confirming the high quality of the RNA-seq data in this study.

### Supplementary information

Table S1

Supplementary figures

## Data Availability

The authors declare that no custom code was used in this study.
