# Supplementary material for: De novo transcriptome assembly database for 100 tissues from each of seven species of domestic herbivore
Source: Sci Data. 2024 May 11;11:488. doi: 10.1038/s41597-024-03338-5 (PMC11088706; doi:10.1038/s41597-024-03338-5)
Supplement: Supplementary file 2 — Supplementary figures [file 41597_2024_3338_MOESM2_ESM.pdf]

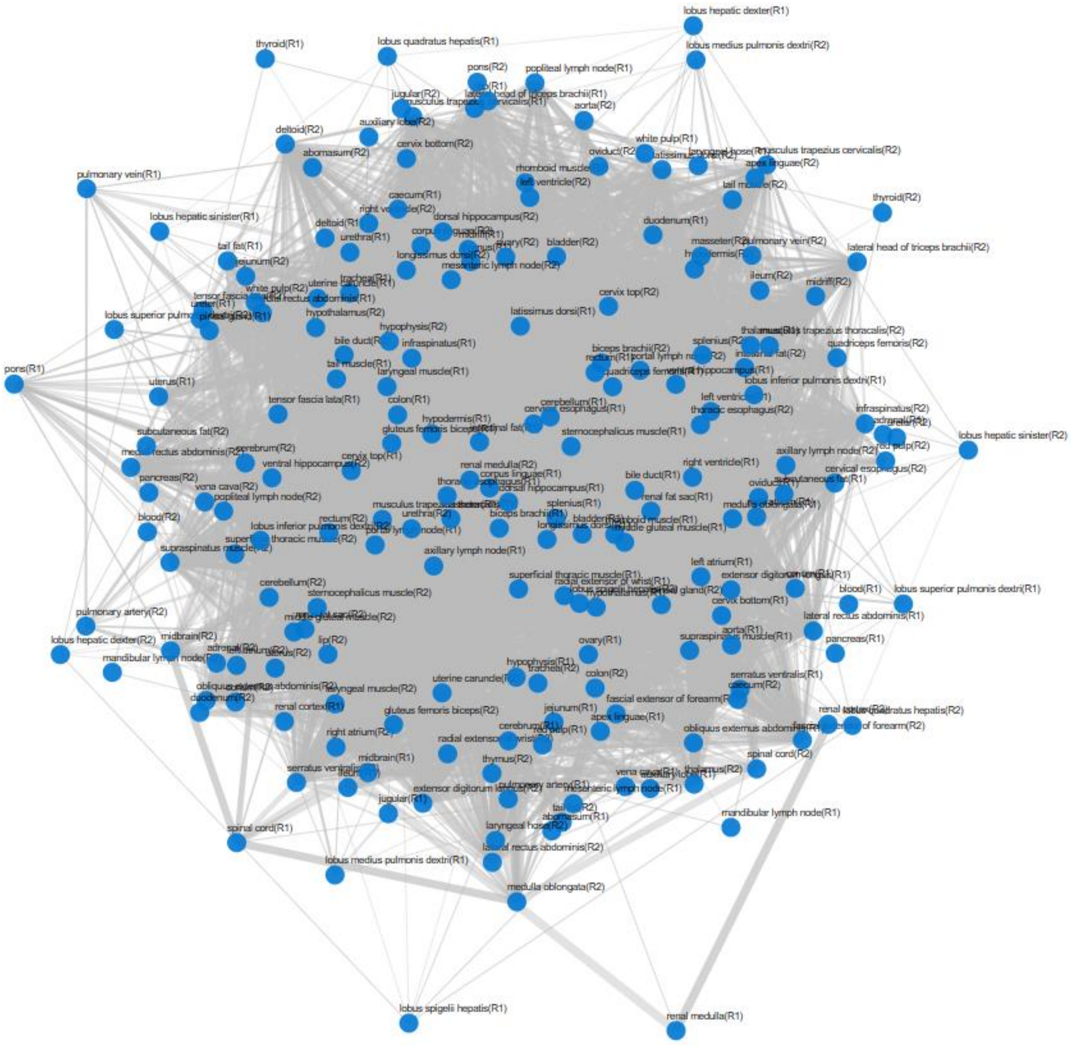

Figure S1 Pearson correlation coefficients analysis on the gene expression of tissues from the Northeast draft horse.

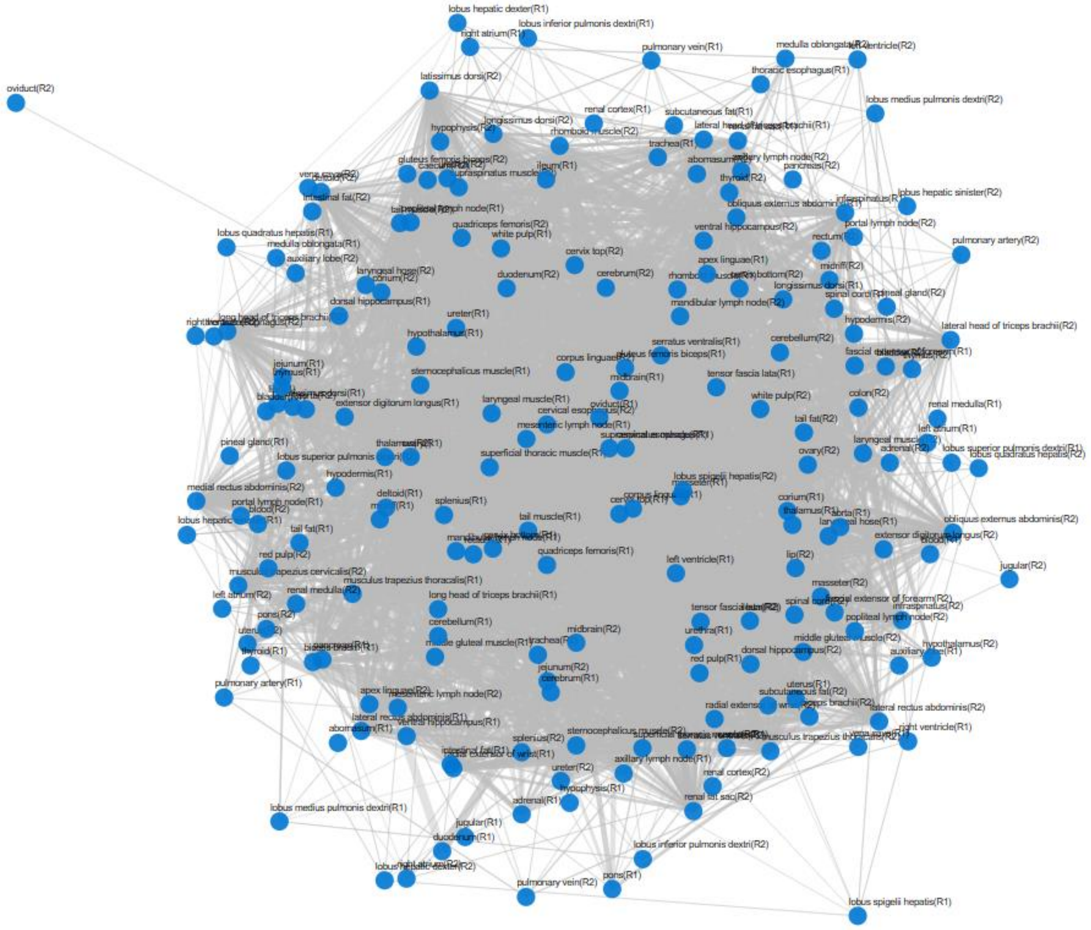

Figure S2 Pearson correlation coefficients analysis on the gene expression of tissues from the Guanzhong donkey.

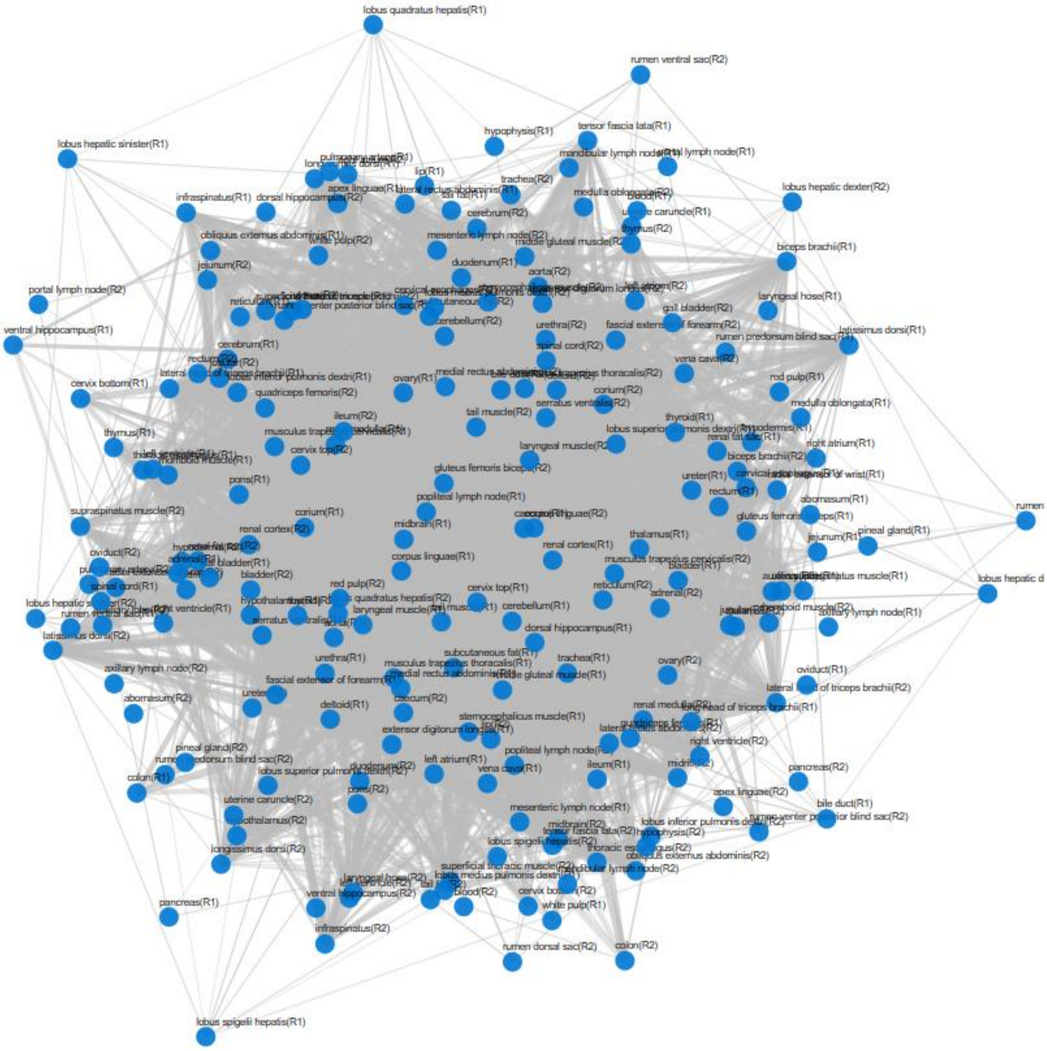

Figure S3 Pearson correlation coefficients analysis on the gene expression of tissues from the Chinese Holstein cattle.

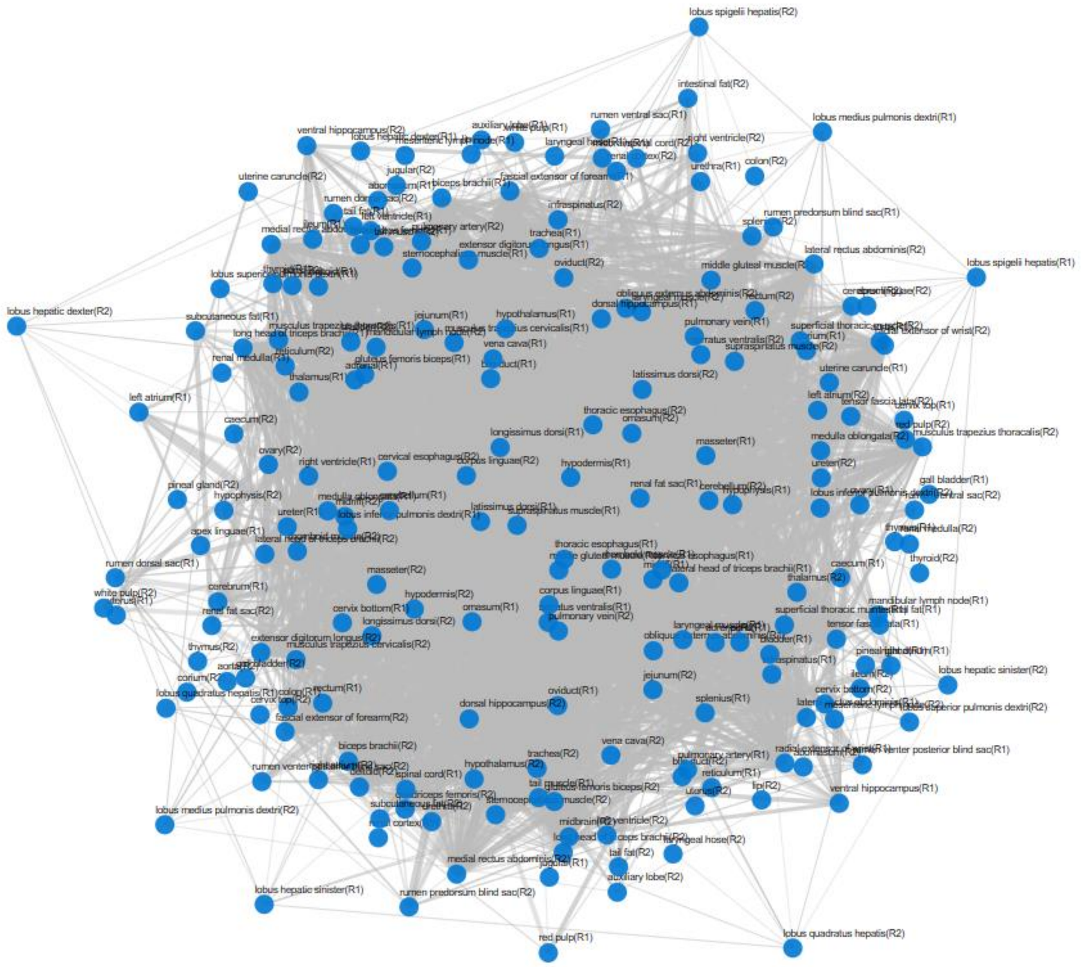

Figure S4 Pearson correlation coefficients analysis on the gene expression of tissues from the Haimen white goat.

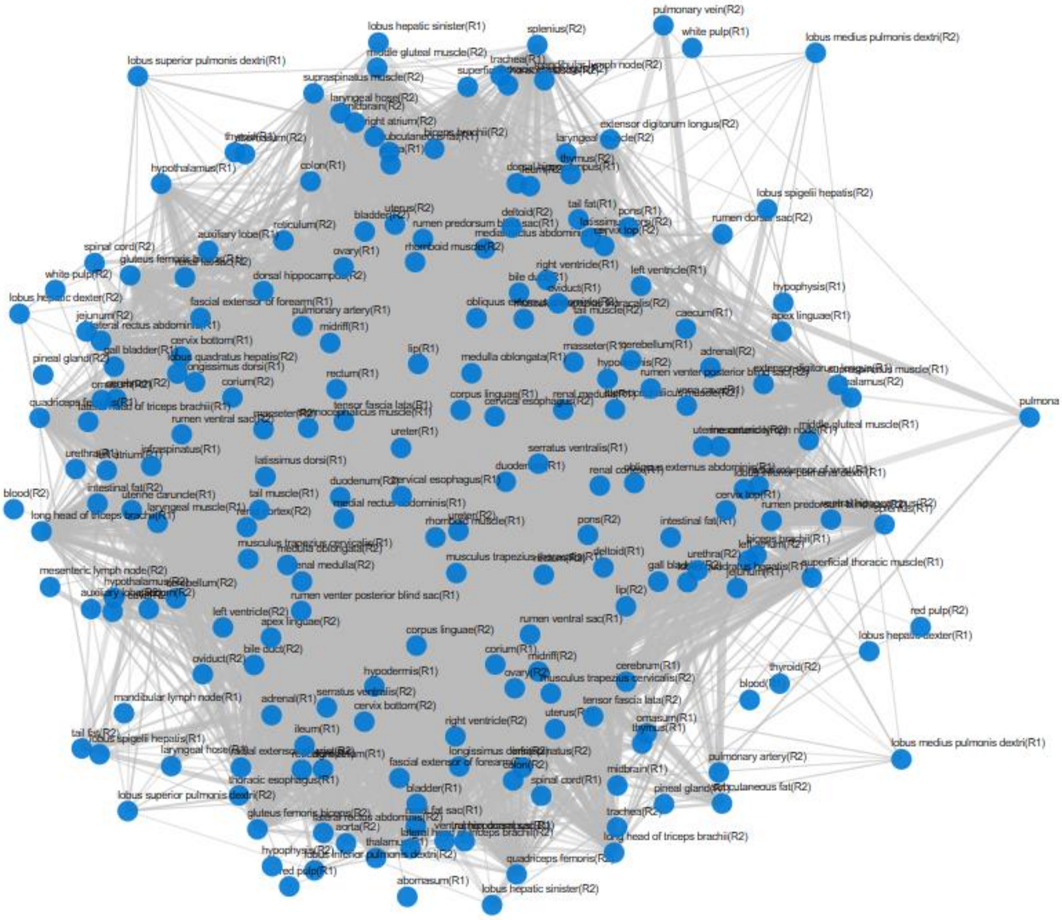

Figure S5 Pearson correlation coefficients analysis on the gene expression of tissues from the Hu sheep and the small-tail Han sheep.

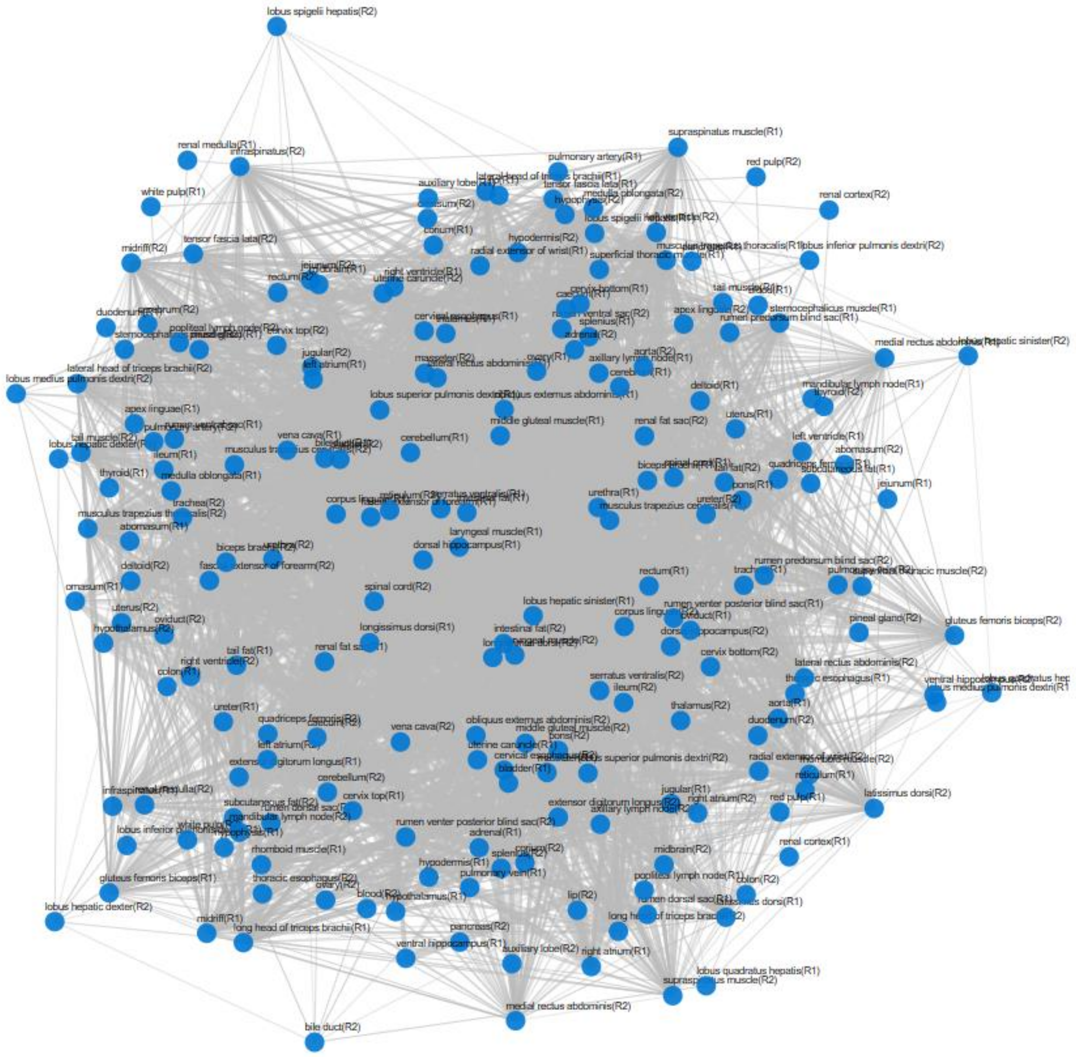

Figure S6 Pearson correlation coefficients analysis on the gene expression of tissues from the sika deer.

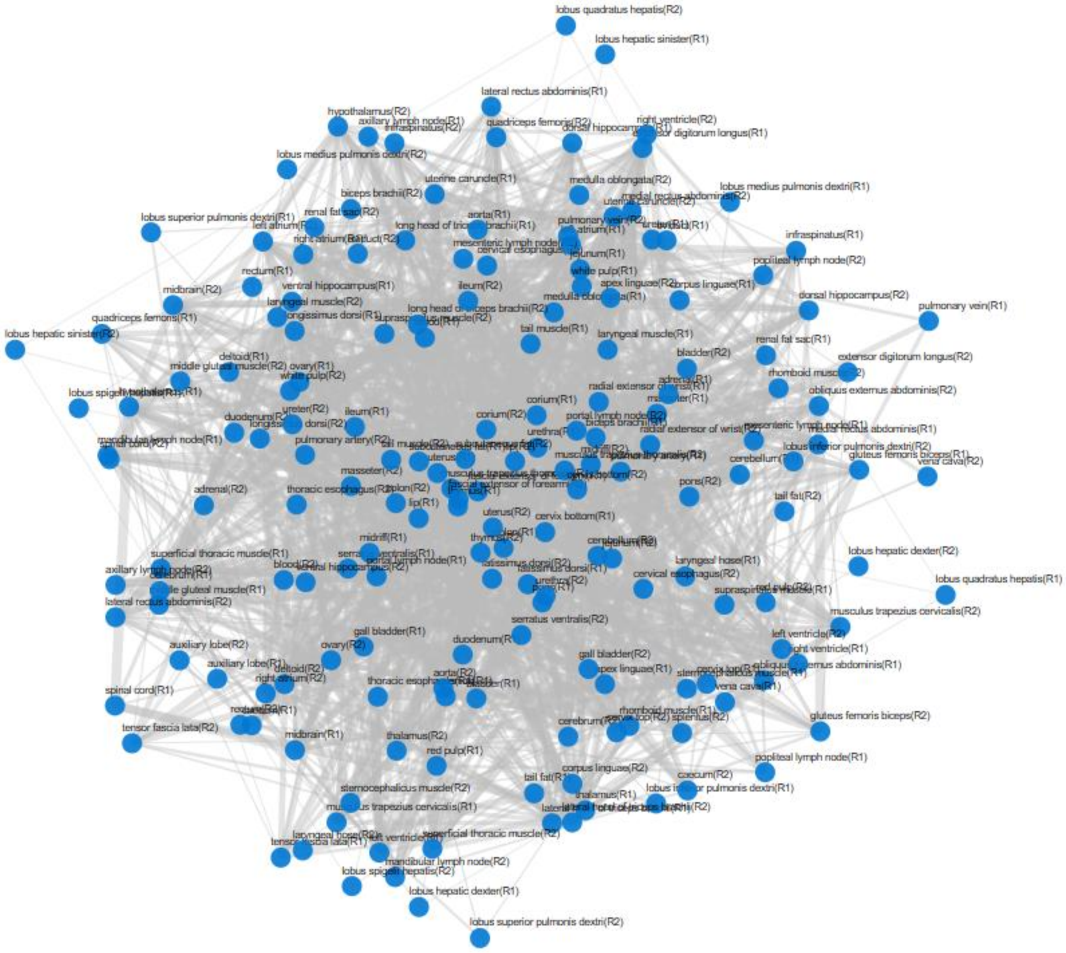

Figure S7 Pearson correlation coefficients analysis on the gene expression of tissues from the New Zealand white rabbit.
